# Utilization of Host and Microbiome Features in Determination of Biological Aging

**DOI:** 10.3390/microorganisms10030668

**Published:** 2022-03-21

**Authors:** Karina Ratiner, Suhaib K. Abdeen, Kim Goldenberg, Eran Elinav

**Affiliations:** 1 Immunology Department, Weizmann Institute of Science, 234 Herzl Street, Rehovot 7610001, Israel; karina.ratiner@weizmann.ac.il (K.R.); suhib.abdin@weizmann.ac.il (S.K.A.); kim.goldenberg@weizmann.ac.il (K.G.); 2 Division of Cancer-Microbiome Research, Deutsches Krebsforschungszentrum (DKFZ), Neuenheimer Feld 280, 69120 Heidelberg, Germany

**Keywords:** microbiome, aging, clocks, biological age, personalized medicine

## Abstract

The term ‘old age’ generally refers to a period characterized by profound changes in human physiological functions and susceptibility to disease that accompanies the final years of a person’s life. Despite the conventional definition of old age as exceeding the age of 65 years old, quantifying aging as a function of life years does not necessarily reflect how the human body ages. In contrast, characterizing biological (or physiological) aging based on functional parameters may better reflect a person’s temporal physiological status and associated disease susceptibility state. As such, differentiating ‘chronological aging’ from ‘biological aging’ holds the key to identifying individuals featuring accelerated aging processes despite having a young chronological age and stratifying them to tailored surveillance, diagnosis, prevention, and treatment. Emerging evidence suggests that the gut microbiome changes along with physiological aging and may play a pivotal role in a variety of age-related diseases, in a manner that does not necessarily correlate with chronological age. Harnessing of individualized gut microbiome data and integration of host and microbiome parameters using artificial intelligence and machine learning pipelines may enable us to more accurately define aging clocks. Such holobiont-based estimates of a person’s physiological age may facilitate prediction of age-related physiological status and risk of development of age-associated diseases.

## 1. Introduction

Microorganisms that reside on and within the human body surfaces constitute a complex ecosystem, termed the microbiome, that is shaped by a partially understood array of ‘host-microorganisms’ and ‘microorganisms-microorganisms’ interactions. Despite the apparent stability of this ecosystem, its composition changes in response to lifestyle, dietary interventions, diseases, and aging [1,2]. During the first three years of life, the human gut microbiome undergoes rapid changes, followed by a long phase of stability during adulthood and, finally, gradual changes associated with aging [1]. This was further validated by various clinical cohort studies that have identified a strong correlation between microbiome composition and age [3,4].

Aging is characterized by gradual functional alterations and susceptibility to aging-related disorders, which may ultimately lead to tissue dysfunction and death [5]. Alterations that are exhibited throughout life, including lifestyle, mobility, nutritional changes, chronic consumption of medications, and change in residential status, may impact the aging process, which in turn impacts life expectancy [6]. This concept is illustrated by the variation in life expectancy across countries [7,8,9,10,11,12]. For example, due to lifestyle-related factors, the average lifespan in the United States is amongst the lowest in the developed world [11]. As part of the physiological changes that characterize mammalian aging, alterations in the gut microbiome composition are gradually observed in aged compared to young individuals, in both humans and rodents [13,14,15]. This is exemplified by gut microbiome changes in composition and function in individuals over 65 years of age [13,16,17], which may result from age-related nutritional, habitual, medication, and other life-style modifications hallmarking the aging process. For example, several studies have revealed associations between elderly gut microbiome composition and measures of frailty [18], physical fitness [19], and diet [20].

In general, the elderly microbiota is characterized by a decline in diversity, expansion of Bacteroidetes at the expense of Firmicutes phyla, an increase in the abundance of opportunistic enteropathogens, and a reduction in species known for producing short-chain fatty acids (SCFAs), in particular butyrate [1,16,17,21,22]. Several studies have shown a connection between dysbiotic aging microbiomes and some physiological aspects of aging, including changes in body composition, impaired immune function, and increased susceptibility to disease, among others [15,23]. Body compositional changes associated with age include an increase in abdominal fat and a decrease in bone density and muscle mass, which can lead to reduced mobility and an increased risk of age-related diseases, including cardiometabolic diseases, sarcopenia, and osteoporosis [24,25,26,27]. A recent study found that a higher abundance of fecal *Christensenellaceae*, *Porphyromonadaceae*, and *Rikenellaceae* was explicitly associated with more favorable body composition in old age, namely lower abdominal adiposity [23]. Future studies on how microbes affect fat distribution in aging are of vital importance, since abdominal adiposity is linked to metabolic syndrome and its associated morbidities [28,29]. The microbiome has also been implicated in changes to bone density in aging, with *Lactobacillus reuteri* in particular able to improve bone mineral density in murine models [30], as well as increases in tibial bone density in elderly women [31], suggesting a therapeutic agent for osteoporosis. Another hallmark of aging is the dysregulated overstimulation of the immune response, leading to a state of chronic systemic inflammation (also known as ‘inflamm-aging’) [32,33]. In mice, the gut microbiome has been linked to chronic inflammation associated with old age [15]. Compared with conventionally raised aged mice, germ-free mice have lower circulating pro-inflammatory cytokines and live longer [15]. Interestingly, transferring germ-free mice into cages containing conventionally aged animals causes an increase in systemic levels of proinflammatory cytokines [15].

The gut microbiome may also contribute to slower aging and longevity. A previous study focusing on extremely old individuals (above 99 years; called centenarians) showed an increased abundance of *Christensenella*, *Akkermansia*, and *Bifidobacterium*, indicating potential life-prolonging effects [34]. It has been postulated that these members of the gut microbiota could play a critical role in protecting against pathogenic infection and other environmental stressors [32,34,35,36,37], and not only as a mere consequence of aging. According to a recent study, the gut microbiome of centenarians contains microbes carrying genes related to bile acid metabolism, including a secondary bile acid with antimicrobial activity against Gram-positive pathogens, suggesting a role in reducing infections among centenarians [38].

Over the last few years, more mechanistic research has aimed to demonstrate how gut microbiomes may play a role in the maintenance of health, longevity, and slow aging in centenarians. In this review, we aim to highlight the importance of proper gut microbiome function in the latter decades of human life, identifying a remarkable signature of longevity, per se, which might constitute a key health indicator of old people that inversely correlates with age-related diseases [34,39].

## 2. The Aging Clocks

Chronological age refers to the number of days, months, and years that a person has been alive, although it may not constitute a trustworthy indicator of a person’s real age, as observed by the significant differences in overall health status and physiological functions of healthy people of a similar chronological age [40]. As a person’s chronological age increases, multiple physical functions may decline and the risk of disease and mortality increases, although these features also display substantial interindividual variability [41,42,43]. Biological age (also referred to as physiological age), which might be lower or higher than the chronological age, describes the real age-related functional status of an individual based on health biomarkers. Aging clocks are means of predicting a person’s biological age based on individualized inputs [44] such as inflammatory profile [45], epigenetic [46], transcriptomic [47,48,49], proteomic [50,51,52], and metabolomic age predictors [53,54]. A useful biological age predictor must be easily collected (and as noninvasive as possible), require low-cost processing, and be dynamically responsive to environmental and medical interventions, which are known to constitute critical factors for determining lifespan and health. Recently, a ‘gut clock’ has been developed for the analysis of the gut microbiota, showing a promising ability to reveal the host’s biological age based on microbiome diversity, taxonomic composition, functional pathways, and metabolomic composition. Intriguingly, some microbiome roles were suggested to be causative, rather than associative, in contributing to aging processes. A recent study revealed that differences in the composition of the gut microbiota of mice after exposure to antibiotics in early life significantly affected immunity, metabolism, and survival [55]. This is consistent with human studies that showed a correlation between short life expectancy risk factors, such as obesity and infections susceptibility, and early life antibiotic usage [56,57]. Similarly, the high rates of uncontrolled antibiotic usage in aged care residents could result in reduced colonization resistance and increased abundance of antibiotic-resistant bacteria, which, in turn, may predispose the elderly to life-threatening infections [58]. Collectively, gut microbial diversity may constitute a predictor of biological age, although its relevance in extreme aging is yet to be elucidated. A better understanding of the roles played by the gut microbiome in the elderly is essential in establishing better predictors of life expectancy and establishing dietary interventions that can impact aging and health status. Mediterranean diet intervention showed promising results, such as reducing frailty and improving health status, through altering the gut microbiome [59]. In the following sections, we will focus on the integration of different facets of host and gut microbiome data in optimizing aging clocks, identifying microbiome-associated targets in delaying the onset of age-related diseases, and the biological aging process in improving life quality and expectancy.

### 2.1. Host-Based Aging Clocks

Over the course of aging, many factors within the host undergo changes at all levels of biological organization, which may serve as biomarkers of aging. Among these host-derived aging biomarkers are epigenetic changes [60,61], low-grade inflammation [45], changes in gene expression [47], protein [50,52], and metabolite [62] levels. Among epigenetic modifications, the DNA methylation at CpG sites is among the most accurate biomarker of aging to date [63]. In 2013, the first epigenetic clocks were described by Hannum [60] and Horvath [61], which used machine learning algorithms utilizing DNA methylation levels in hundreds of CpG sites to predict age. Studies have demonstrated that epigenetic age is able to predict all-cause mortality better than long-established risk factors and chronological age [64,65]. Although DNA-methylation-derived epigenetic clocks have excellent predictive power for age and mortality [66], they feature weak associations with specific outcomes related to age, including heart and brain diseases [67,68,69]. The development of high-throughput omics technologies (genomics, transcriptomics, proteomics, and metabolomics) has led to a wealth of data that can be utilized to define new aging clocks beyond the previously described epigenetic clocks.

One such example is the inflammaging clock [45]. Inflammaging is the sterile, chronic, low-grade systemic inflammation that develops during aging [32,33]. Indeed, chronic inflammation has strong links to the leading causes of death, such as cardiovascular disease, cancer, and metabolic diseases [70,71,72]. Alpert et al. [73] profiled the transcriptome, immunome, and cell subset frequencies of 135 adults longitudinally to construct a trajectory of immune aging (IMM-AGE) that accurately predicted all-cause mortality better than chronological age. More recently, Sayed et al. [45] developed an inflammatory age clock (iAge) by using deep learning methods on blood immune biomarkers of 10,001 individuals. iAge can predict multiple disease phenotypes across cohorts and can be used as a metric for healthy versus unhealthy aging. Transcriptomic aging clocks have been based on gene expression in a tissue-specific manner [47] across multiple tissues [74,75] and in blood [76]. However, transcriptomics-based aging clocks are not as accurate as other methods (such as DNA methylation) due to high variability in the data, study parameters, and data analysis. To overcome this variability, Meyer et al. [49] used a model organism (*Caenorhabditis elegans*) to define a gene set that predicts biological age with high accuracy. Their binarized transcriptomic aging (BiT age) clock could be applied to predict biological age in humans too. Like the change in the transcriptome with age, the proteome shows distinct changes too, and a novel proteomic aging clock has been recently described [50]. Of particular relevance is the metabolomic clock, which can be used to both predict biological age and identify systemic biomarkers that strongly correlate with increased age. Metabolites represent the final products of cellular metabolism and provide a more complete picture of biological processes. Robinson et al. [62] developed a model of age using untargeted metabolomic profiling of serum and urine. They found that while metabolomic age acceleration was not associated with epigenetic age, it did correlate with mortality risk factors, including obesity, type 2 diabetes, alcohol abuse, and depression [62].

Omics technologies and access to large data sets have led to the development of multiple types of host clocks that attempt to model biological age. However, these clocks fail to take into consideration the critical role of the microbiome and its proven links to epigenetic regulation, immune activity, the transcriptome, and the metabolome. By defining microbiome-based clocks and integrating them with host measures, more precise pre-dictions of age can be developed.

### 2.2. Microbiome-Based Diversity Clock

**Figure 1:**
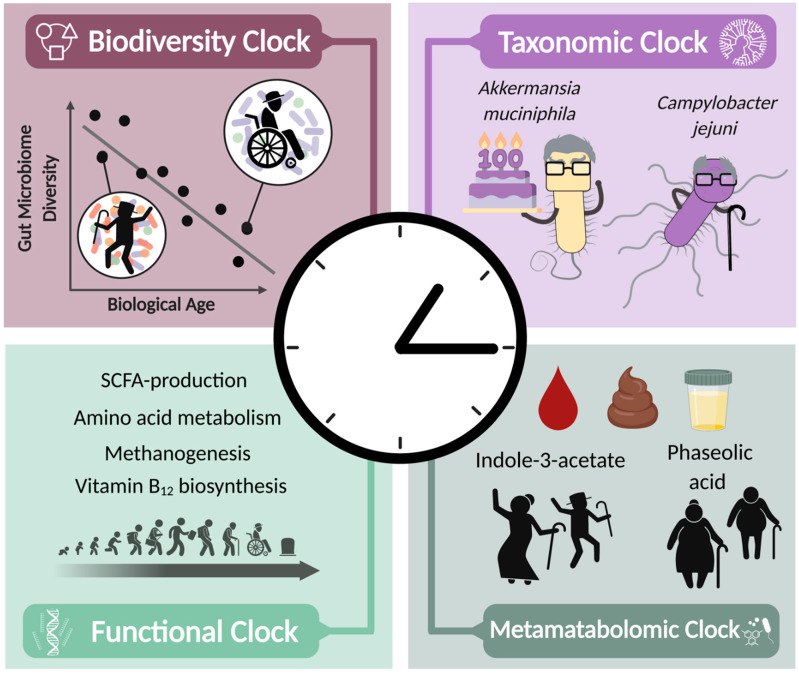
Four microbiome-based aging clocks. Age-related decline in gut microbiome biodiversity can be used to determine biological age. Certain species are enriched in different individuals and relate to the biological clock; for example, *A. muciniphila* is enriched in centenarians and is associated with longevity, while *C. jejuni* is enriched in individuals with higher biological age. Metagenomic and metatranscriptomic analysis gives insights into microbiome functions that affect host aging. Metametabolomics of blood, urine, or stool can identify microbiome-derived metabolites associated with biological age. SCFA: short-chain fatty acids. Figure created with BioRender (biorender.com).

Microbiome diversity, which can be determined using DNA-based next-generation sequencing approaches, including sequencing of the ribosomal 16s rRNA gene (16s rRNA sequencing) or whole shotgun metagenome sequencing, reflects the number of different species and their relative abundance (richness and evenness) in a given microbial ecosystem or between ecosystems (known as α-diversity and β-diversity, respectively). In 2015, O’Toole et al. [77] reported an association between the loss of diversity in the core microbiota groups and an increased frailty index, which is considered a quantitative measure of biological age. Other studies have confirmed increased gut taxonomic α-diversity is associated with healthier aging and increased longevity [78], while decreased gut microbiome diversity has been associated with hospitalization [20]. Notably, several studies have shown that α-diversity, or more precisely microbial richness, is negatively correlated with the frailty index (biological age), but not with chronological age [18,79] (Figure 1). Altogether, these studies point to the possibility that gut microbiome diversity could function as a biological clock.

Sala et al. [80] proposed a diversity-based model for estimating healthy aging, the ‘Hybrid Niche Nature Model’, in which they incorporated Hubbell’s diversity index, a measure that focuses on species that are rare and most abundant, instead of traditional methods for richness and evenness (e.g., Shannon or Simpson indices). Analyzing 1649 stool samples from six 16S rRNA sequencing data sets, representing a wide range of dietary habits, health status, ages (0 to 109 years), and nationalities, showed an increase in microbial diversity in healthy aged individuals, while decreased microbial diversity was associated with unhealthy phenotypes at older ages. Based on data obtained from the ELDERMET study (the most comprehensive study of microbiome data from older people to date), the model was suggested to constitute a good predictor of the health status in aged individuals, even more so than the commonly used diversity quantification methods, such as Shannon, Simpson, Pielou, and Hill’s indices [80]. Although the gut microbiome α-diversity is significantly associated with health and age, centenarian microbiomes vary widely across data sets and geographical locations, limiting the ability to draw universal conclusions about this important group [78,80,81,82,83]. More recently, Wilmanski et al. [84] examined more than 9000 people aged 18–101 years and found that the composition of the gut microbiome of people in midlife becomes increasingly individualized as they age (determined by Bray-Curtis uniqueness, a measure of β-diversity). People with a more individualized microbiome had better clinical laboratory values (e.g., lower triglyceride levels), required fewer medications, and had better physical health and greater mobility. In people over 84 years of age, this ‘uniqueness’ appears to be associated with longer life expectancy [84]. This gradual increase in the uniqueness of the gut microbiome is accompanied by clear microbial metabolomic signatures in plasma [84], related to amino acid metabolism, which have already been mentioned in studies of centenarians (see further in Section 2.5). This may mean that the longitudinal β-diversity analysis can resolve the inconsistencies observed in the α-diversity of centenarians across different data sets and provide a more accurate biological age prediction. Altogether, these findings highlight that the microbiome community structure, namely diversity, could serve as an indicator of biological aging and life expectancy.

### 2.3. Microbiome-Based Taxonomic Clock

Defining the constituents of a given microbiome ecosystem at the genus, species, and strain level enables the association of discrete community members with a second microbiome-aging clock, the taxonomic clock. Several studies, which have examined the relationship between the microbiome and aging, identified taxa that are associated with aging and aging-related diseases. For example, Maffei et al. [79] reported the existence of differentially abundant genera, including *Eggerthella*, *Ruminococcus*, and *Coprobacillus* genera, among different groups who were classified based on frailty index. A recent study evaluated the ability of oral, gut, and skin microbiomes to predict adult age based on 16 s rRNA sequencing data of healthy individuals collected from multiple publicly available studies [85]. Among the three body-site-specific microbiome signatures, the skin microbiome was the most accurate in predicting chronological age, with a mean absolute error of 3.8 years, while the gut microbiome was the least accurate, with a mean absolute error of 11.5 years [85]. Further studies involving populations with background diseases are needed to determine the relevance of model prediction of biological age rather than chronological age. Of note, the aforementioned study relied on 16s rRNA rather than whole genome sequencing, which has the disadvantage of being unable to characterize bacterial composition at the species level thoroughly and unbiasedly [86]. Consequently, it is not possible to conclude from this study whether bacteria residing in the non-gastrointestinal tissues, namely the oral cavity and the skin, provide better biological age predictions than those found in the gut. This merits future studies.

In another recent study, Galkin et al. [87] generated a machine learning model of aging clock, using metagenomic data that were investigated in 13 published studies, including more than 4000 metagenomic samples of 1165 healthy individuals aged 18–90 years. Their most accurate prediction achieved a mean absolute error of 5.91 years and included both well-studied participants of the gut community (e.g., *Bifidobacterium* spp., *Akkermansia muciniphila*, *Bacteroides* spp., *Escherichia coli*), and rarely-described species such as *Streptococcus equinus*, or *Ornithobacterium rhinotracheale*. The fact that unusual bacteria contributed to the model opens up new possibilities for looking for novel bacteria that may be involved in aging. In such taxonomic clock model, particular microbes may influence model predictions to accelerate or delay the determined biological age. For instance, the clock projected that an individual with a high abundance of *Campylobacter jejuni* is classified as being older than his chronological age (Figure 1). *C. jejuni* infection is the most common cause of gastroenteritis and associated with Guillain-Barré syndrome [88], and can activate innate immune pathways in a variety of means though the formation of inflammasomes [89]. Meanwhile, the bacterium *A. muciniphila* modifies the taxonomic clock towards a biological age that is younger than the chronological age [87]. In addition to bacteria, the methanogenic archaea *Methanobrevibacter smithii*, which removes diverse bacterial end products of fermentation, also shifted to a younger predicted age [87]. Interestingly, compared with younger individuals, centenarians show an increase in both *M. smithii* and *A. muciniphila* [35,38] (Figure 1). The taxonomic clock emphasizes biological age and not chronological age as the determinant of health status, so that people with type 1 diabetes are considered older as a group compared to their chronological age [87]. The gut microbiome has been strongly implicated in obesity, and has a characteristic signature [90]. Furthermore, obesity is associated with accelerated aging and a myriad of aging-related diseases [91]. Utilizing BMI or fat mass as an additional parameter when developing aging microbiome clocks will aid in identifying individuals at greater risk for age associated diseases. Of note, validating the taxonomic clock in populations not presented in the aforementioned study may further enhance its accuracy across geographical locations [92,93].

### 2.4. Microbiome-Based Functional Clock

A third category of microbial aging clocks is based on characterization of microbial genes and their functional features (Figure 1). Unlike microbial taxa, this aging clock has the advantage of being consistent across cohorts rather than having limited reproducibility, as microbiome functions represent a better ‘common denominator’ feature of healthy status as compared to the highly variable microbial consortia that contribute these functions in different individuals [94]. When characterized at the DNA level, microbial function describes the microbial capability or potential to generate a function, such as producing, degrading specific molecules, and other enzymatic activities. A more straightforward assessment of microbial function requires interrogation at the mRNA level (metatranscriptomics) or the small-molecule (metabolomics) and protein (metaproteomics) level.

Indeed, using human metagenomic data and a supervised machine learning algorithm, Lan et al. [95] showed alterations in gut microbiome function that are associated with aging. These included decreased vitamin B12 synthesis, reduced reductase activity, increased DNA damage, stress, immune system impairment, and upregulated glycosyltransferases. Using a metatranscriptomic analysis pipeline, Gopu et al. [48] suggested that microbial expression patterns could be used to predict biological age. By analyzing meta-transcriptomic profiles of approximately 90,000 individuals, aged between 0 and 104 years old, with a wide array of lifestyle habits and disease states (e.g., digestive and metabolic disorders), a strong positive correlation was identified between the microorganisms’ functional profiles, which are related to methanogenesis pathways, and age. Several metabolic pathways were negatively associated with aging, including SCFA production, vitamin B12 biosynthesis, and amino acid metabolism [48] (Figure 1), probably stemming from microbial taxonomic changes. The metatranscriptomic clock also revealed that people on a vegetarian diet tend to be biologically younger than those on a Paleo diet or those with irritable bowel syndrome (IBS) [48]. As with other aging clocks, this method provides an example of how lifestyle and health status influence biological age.

### 2.5. Metametabolomic Clock

Metabolomic profiling involves the identification and quantification of metabolites that are present in cells, tissues, and biofluids, including microbially derived compounds. Metabolomic profiling might constitute a promising tool to determine a person’s biological age, as metabolic changes were shown to occur along with aging, possibly reflecting changes in biological functions of both the host and microbiome [96]. While predicting the biological age by analyzing bacterial metabolites is still in its infancy, emerging pieces of evidence show that circulating metabolites can help establish an aging clock [53,54,62,97]. For example, using plasma metabolomic profiles of individuals between 18 and 80, Johnson et al. [96] identified 21 metabolites that are associated with biological age. Interestingly, several of these molecules were previously identified as microbe-associated metabolites [98,99,100]. Of note, higher concentrations of putrescine (a type of polyamine) and indole-3-acetate were associated with a lower biological age [96] (Figure 1). Conversely, higher concentrations of phaseolic acid have been observed in biologically older individuals [96] (Figure 1), despite its previously proposed antiaging function [101]. Therefore, the results of this study suggest that microbial metabolites, together with the hosts’ metabolites, may contribute to establishing an aging clock. Furthermore, the microbial uniqueness described by Wilmanski et al. [84] (see above in Section 2.2) has also been associated with several microbiome-derived metabolites in plasma, including protein-bound uremic toxins, such as 3-indoxyl sulfate, 6-hydroxy-indole sulfate, indole acetate, indole propionate, p-cresol sulfate (p-CS), p-cresol glucuronide, and phenylacetylglutamine (PAG) [84]. Results of this study indicate that age-related changes in the gut microbial amino acid metabolism are reflected in plasma metabolomes and could have a predictive value for mortality [84].

Secreted metabolites in urine and feces have also been investigated in the context of aging clocks. For example, several gut-derived metabolites in the urine have been reported to correlate positively with age, including PAG [102], 4-cresyl sulfate [102], and trimethylamine-*N*-oxide [97,103]. Furthermore, centenarians have higher levels of p-CS and PAG in their urine [104], consistent with findings from the plasma metabolome [84]. In addition to urine, the fecal metabolome could probably serve as an aging clock. For example, fecal levels of SCFAs, such as propionic and butyric acids, decrease with age, whereas branched SCFAs such as isobutyric and isovaleric acids and ammonium are increased in centenarians [38]. A recent study showed that the microbially modified secondary bile acids, including isoallo-lithocholic acid and other lithocholic acid isoforms, are abundant in high concentrations in the feces of centenarians [38].

In parallel to advances in metabolomics, proteomics analyses have become increasingly useful in understanding protein composition and its function in different health conditions and can explain the differences in the metabolome. To date, there has been no comprehensive bacterial proteome-based aging clock, probably due to the lack of standardized protocols for both protein extraction and data analysis. However, altered human and microbial proteomes in obese and type 1 diabetes, two health risk factors, have been reported [105].

### 2.6. Integrated Data Sets in Predicting Biological Age

Given the complexity and heterogeneity of biological aging, it is obvious that setting an accurate microbiome-based aging clock can be accomplished only by integrating different types and sources of data, and that new computational methods are required. The first attempt at age prediction using multiple microbial omics was made in a recent study that integrated taxonomic and functional information from stool metagenomics [106]. In this study, Chen et al. [106] developed a multiview ensemble machine learning method for age prediction from metagenomic sequencing data using almost 4500 stool samples collected from 31 cohorts from 28 different countries (including Europe, America, Asia, Africa, and Oceania). After considering the influence of geographical factors, the results show that the model based on the combination of microbe species and their functional pathways provided the best prediction of chronological age (with an average mean absolute error of 8.33 years; R^2^ = 0.599). While aging clocks are designed to provide accurate predictions of host aging, the main advantage of such an integrated model may not be its greater accuracy but its ability to expand our understanding of aging. Therefore, combining microbiome-related data sets, such as diversity, taxonomic, functional, and/or integration of data sets, of both the host and the microbiome may offer a comprehensive and more accurate aging clock.

Several studies showed the benefit of combining multiple biomarkers for biological age prediction, although they are all host-related biomarkers [107,108,109,110,111]. The epigenetic clock is considered as the most precise representation of aging decline over time, while being less sensitive to lifestyle factors [112]. On the other hand, the gut microbiome is highly affected by behavioral, lifestyle, environmental, and interventional factors [113]. Therefore, the combination of host- and microbiome-derived aging clocks holds great potential for reflecting precise and accurate biological aging. However, data integration must be treated carefully to overcome statistical complexities. This can be realized through the use of multilayered networking for connecting different types of information [114], such as tensor-based approaches for discovering patterns in data [115]. Other machine learning approaches were also powerful in reanalyzing large data sets, enabling exceptionally high levels of data integration and providing novel insights [116].

## 3. Perspectives, Opportunities, and Challenges in the Research of Microbiome Aging Clocks

### 3.1. Implication of Host and Microbiome Features in Determination of Age-Associated Diseases

The biological age of a host is ultimately linked to its background of disease status. When biological age is higher than chronological age, a person is more likely to develop a disease. On the other hand, given that individuals with diabetes and IBS display accelerated biological aging in comparison to people without these diseases, as was demonstrated by the taxonomic and metatranscriptomic clocks [48,87], an individual’s health and disease status may accelerate biological age prediction. Research on microbiome-based clocks has typically focused on determining an individual’s biological age, which does not directly address the various comorbidities of aging; however, the findings on diabetes and IBS patients provide the first indication that biological age might also be compromised in populations with other diseases associated with aging (such as Alzheimer’s disease, dementia, osteoporosis, cancer, hip fractures, etc.). While this has not yet been proven or tested, the observations described above may represent only the tip of the iceberg when it comes to the application of these clocks to disease management. Many questions remain, such as: Is it possible to identify people at a high risk of developing age-related diseases by measuring their biological age? Is it possible to predict specific diseases?

As for host-based markers, there have been attempts to tailor multiple measurements to predict the risk of specific age-related diseases, including cardiovascular diseases [67,117,118], type 2 diabetes [119,120], neurodegenerative disorders [52,121], and cancer [122,123]. While there is controversy regarding the ability of the DNA-methylation-derived epigenetic clocks to predict cardiovascular outcomes [67,117], measures of post-translational modifications by arginine methylation in human hair proteins have been suggested to be useful in that regard [118]. Despite the intriguing results and advantages of the noninvasive sampling method, this method does not appear to be better than existing ones and is, in any case, not suitable for the elderly suffering from baldness. For metabolic disease prediction, the DNA methylation age of blood may be suitable to predict age-related type 2 diabetes risk [119]. Epigenetic biomarkers may reflect age-related DNA methylation changes in pancreatic cells and are associated with insulin secretion in vivo and type 2 diabetes [120]. Lehallier et al. [52] found that people predicted to be younger than their chronological age based on a proteomic aging clock performed better on physical and cognitive tests. Another study constructed age predictive models using targeted and untargeted metabolomic and lipidomic profiles of cerebrospinal fluid from healthy individuals and found an increase in prediction error when tested on individuals with Alzheimer’s and Parkinson’s disease [121]. As for cancer, it was shown that blood methylation age has been shown to predict future lung cancer incidence [122] and may reflect epigenetic changes related to cancer development that may serve as minimally invasive biomarkers for early cancer diagnosis [123].

As for the microbiome, numerous studies have linked gut microbiome dysbiosis to various physiological phenotypes, ranging from risk-related conditions to diseases [124,125]. It has been suggested that identifying microbial taxa associated with the host disease may benefit early diagnosis and prognosis [126]. Indeed, over the past years, several studies have shown that specific diseases and health-related conditions can be predicted using microbiome data [127,128,129], including cancer [130], type 2 diabetes [131], and Parkinson’s disease [132]. These, however, are not necessarily related to aging. According to a recent study, in order to improve disease prediction, it is important to consider the chronological age of an individual [133]. There is still a need for more research to investigate the exact microbial signatures linked to diseases associated with aging. Beyond diagnostic and prediction of diseases, the gut microbiome data could also be useful in the prediction of responsiveness to treatment in multiple age-related diseases. For instance, a recent prospective pilot study has shown that the microbial taxonomic composition of elderly with geriatric depression may be indicative of their response to antidepressants [134]. In another study, the baseline composition of the fecal microbiome, particularly the relative abundance of *Faecalibacterium prausnitzii*, was used to predict whether Crohn’s disease patients would experience a clinical relapse following discontinuation of immunosuppressive therapy [135].

### 3.2. Challenges in Microbiome Aging Research

The gut microbiome databases constitute a massive usable source for establishing tools for predicting biological age and mortality. However, there are several technical and conceptual obstacles that still need to be carefully handled. This is exemplified by the inability to generalize conclusions due to differences between geographical and demographical groups. When studying how gut microbiomes differ across populations, Yatsuneko et al. [136] found pronounced changes in gut bacterial composition and functional genes between the US population and those from Venezuala and rural Malawi. Other large-scale clinical studies found that taxonomic differences in the microbiome could be explained by ethnicity [137] and geography [92]. Indeed, these differences are also seen in aged individuals and centenarians from different parts of the world. For example, centenarians in an Italian cohort had a microbiome specifically enriched in *Akkermansia*, *Bifidobacterium*, and *Christensenella* [34], compared to centenarians in the Chinese Hainan Centenarian Cohort Study, which was dominated by *Bacteroides* and *Escherichia* [138] (reviewed in detail [83]). With increasing awareness of the differences between populations, more studies involving diverse ethnicities and regions have been conducted, but there still are remaining uncertainties. First, many of the microbiome-based aging clocks discussed above are based on studies with varying sample sizes, which leads to biased results when interpreting results with regard to the general world population. Second, despite the general assumption that more diverse populations and larger sample sizes can provide insights that are relevant to the world population, it is still unclear how such heterogeneous data should be handled. It has been suggested, for example, by Chen et al. [106], that geographical subregion information could significantly improve model age prediction performance. Third, it is important to recognize that in addition to the microbiome, aging rates and life expectancy also vary between ethnic and geographic groups [139,140,141]. It has been suggested that regional differences in dietary habits could be important for such variations [83,141]. Therefore, it might be critical to understand first what derives geographical and ethnical differences. In addition to the lack of availability of some data sets and resources, the differences in the methods of acquiring and analyzing data (e.g., extraction or sequencing methods) make it difficult to compare data sets from different studies and geographical regions. Moreover, defining a normalization method is important to distinguish between a real signal and background. However, establishing a universal normalization pipeline might be difficult to realize. Integrating different data sets, including data of both the host and microbiome, might be challenging due to mathematical and statistical complexities, in addition to difficulties in obtaining multiple data sets from the same individual/sample. These constitute exciting avenues of research in years to come.

### 3.3. Outlook

Estimating real biological age may enable us to better predict the changes hallmarking healthy and unhealthy aging processes. An increasing number of aging clock inputs, models, and pipelines are being explored to achieve this formidable task, including epigenetic and inflammatory clocks [45,46]. There are various factors that influence the microbiome and the aging process, including intrinsic factors (genetics, gender, and ethnicity) as well as extrinsic factors (demographic, geographical, diet, physical activity, drugs, smoking, and others) [142,143,144,145,146]. Recent advances in microbiome research, including the ability to define the microbiome components and functions and the realization that microbiomes may contribute to aging-related processes, have led to the inclusion of microbiome-based features in aging clocks. However, interindividual microbiome differences as well as demographical, geographical, and dietary impacts constitute challenges in integrating the microbiome in personalized aging models. One way to overcome this variability is by using a relatively large sample size; this lends more power to the age prediction model and minimizes the relative signal from background noise. Another way to ensure the model is robust is to test the generalization ability of the model through building it on one data set and then testing it on a novel data set that represents a different population, demographic group, etc.

Currently, most microbiome-based aging clocks consist of only one or two measurements, making it unlikely that they can account for all complex aspects of the aging process. The advantage of big data analysis pipelines in the creation of these different aging clocks provides a foundation on which to identify biomarkers, patterns, and measures that can indicate a personal biological age or risk for age-associated diseases. By integrating both insights from the host’s aging clock and that of the microbiome, a more holistic measure can be developed, which will have more power to predict a personal status. We have discussed the various uses of host and microbiome features in the identification of biological aging and associated threats. However, it is important to note that, in addition to bacteria, other microbiome members, such as fungi, archaea, and viruses, are also potential aging biomarkers. Furthermore, although most studies to date have focused on the gut microbiome, there is evidence that microbes outside the gastrointestinal tract are better predictors of chronological age (e.g., the salivary and skin microbiome [85]), although this has never been tested on the microbiomes of the reproductive organs, lungs, nose, teeth, etc. Optimizing these inputs, integrating nonbacterial and nongastrointestinal microbiome data, and combining microbiome-based data sets with those collected from the host may enable the development of a ‘holobiont clock’ to more accurately predict biological age and associated health ramifications.
